# Predictors of Mortality in COVID-19-Positive Patients On and Off CPAP: A Review From a Tertiary Care Setting in the NHS

**DOI:** 10.7759/cureus.19762

**Published:** 2021-11-20

**Authors:** Furqan Rabbani, Mir Azam Khan, Syed Kashif Kalam, Sudeep Shrestha, Khalid Rashid, Farrukh Ansar, Fahad Ahmad, Hamza Amin, Mustafa Javaid, Anas Al-Fahad

**Affiliations:** 1 Respiratory Medicine, The James Cook University Hospital, Middlesbrough, GBR; 2 Respiratory Medicine, Sunderland Royal Hospital, Sunderland, GBR; 3 Respiratory Medicine, University Hospital of North Tees, Stockton-on-Tees, GBR; 4 General Internal Medicine, The James Cook University Hospital, Middlesbrough, GBR; 5 Medicine, Northwest School of Medicine, Khyber Medical University, Peshawar, PAK; 6 General Surgery, Khyber Teaching Hospital, Peshawar, PAK; 7 General Internal Medicine, Good Hope Hospital, Birmingham, GBR; 8 General Internal Medicine, Kuwait Teaching Hospital, Peshawar, PAK

**Keywords:** covid-19, sars-cov-2, predictors, mortality, cpap

## Abstract

Introduction

Since the first description of a coronavirus-related pneumonia outbreak in December 2019, the virus SARS-CoV-2 that causes the infection/disease coronavirus disease 2019 (COVID-19) has evolved into a pandemic, and as of today, millions have been affected.

Objectives

Our aim was to identify the predictors of mortality in COVID-19-positive patients on or off continuous positive airway pressure (CPAP).

Methodology

This was an observational study. Data were collected from February 2020 to April 2020 with patients admitted to the COVID-19 ward at The James Cook University Hospital, Middlesbrough, England. The inclusion criteria were COVID-19-positive patients confirmed through PCR tests on or off CPAP. Patients who had negative RT-PCR for COVID-19 and those who were intubated were excluded.

Results

A total of 56 patients diagnosed with COVID-19 (through RT-PCR) were included in the final analysis, among which 27 were on CPAP, while 29 did not require CPAP (NCPAP). The overall mean age of the patients was 66 ± 14 (range: 26-94) years. The mean age of CPAP and NCPAP patients was 63 ± 15 (range: 26-85) years and 68 ± 13 (range: 40-94) years, respectively. The ethnicity of 54 (96.4%) patients was White-Caucasian, while 2 (3.6%) were British-Asian. In the study sample, 16 (28.6%) patients expired, of which 11 (40.7%) were on CPAP, while 5 (16.7%) did not require CPAP during the disease course. Correlation analysis showed that overall higher age, Medical Research Council Dyspnoea (MRCD) score, performance status (PS), and consolidation affecting more than one quadrant of the lungs were significantly correlated with increased mortality. Among patients receiving CPAP, higher age, MRCD score, and PS were significant predictors of mortality. Among the NCPAP group, advancing age, respiratory rate, MRCD score, PS, increased creatinine levels, and consolidation affecting more than one quadrant of the lungs were the predictors of mortality.

Conclusion

Even with a small sample size, we can see that there are definitive predictors that are directly proportional to increased mortality in COVID-19 patients on CPAP, such as higher age, performance status, MRCD score, and increased lung involvement of consolidation in more than one quadrant, which can help us rationalize management.

## Introduction

The outbreak of coronavirus disease 2019 (COVID-19) is the most devastating pandemic of the 21st century [1]. This pandemic has threatened other sectors such as global trade and economic development in addition to the health of the general population globally [2]. Patients who are severely affected are often more difficult to treat and have a poor prognosis [3]. While researchers have made rapid progress in understanding the occurrence, progression, and treatment of the disease [4,5], it is also important to identify the risk factors for severe illness from COVID-19.

An earlier retrospective study by Guan et al. [3] of 1099 confirmed COVID-19 cases collected from 552 hospitals and 30 provinces in China found that the highest percentage of patients had a fever (87.9%) or a cough (67.7%), and the least common clinical manifestations were diarrhea and vomiting (<5%). In some patients, the virus may lead to severe complications such as pneumonia, sepsis, acute respiratory distress syndrome (ARDS), hyperinflammation, neurological symptoms, or multisystem organ failure [6].

During the coronavirus disease 2019 (COVID-19) pandemic, Piedmont, together with Lombardy, Emilia-Romagna, and Veneto, was one of the most affected Italian regions. Due to the exceptional demand on intensive care unit (ICU) resources, hospitals increased the number of ICU beds and converted many general wards in respiratory intermediate care units (RICUs) to treat patients with severe pneumonia and ARDS needing respiratory support and monitoring. Indeed, NIV in patients with different therapeutic indications, i.e., full treatment and do not intubate (DNI) [6], has been shown to be successfully applicable outside the ICU [7,8] when appropriate monitored settings and trained personnel are employed.

Continuous positive airway pressure (CPAP) ventilation has been commonly used in the management of COVID pneumonitis patients in the UK. CPAP has been shown to reduce the need for ventilation in patients with COVID pneumonitis with oxygen [9]. Our study aimed to identify the demographics and predictors of mortality of COVID-19-positive patients on CPAP.

## Materials and methods

Our study is a single-center retrospective observational study. Our team collected data with a self-structured questionnaire, which was initially pilot tested, and required amendments were made. This was initially pilot tested on three patients, and it was found that our questionnaire was missing data regarding performance status (PS) and ethnicity, which were essential variables contributing to mortality in COVID-19. Therefore, these variables were added before the full data collection process.

We examined the patients' electronic notes, blood tests, and chest X-ray reports retrospectively. All patients admitted to the respiratory unit with positive RT-PCR for COVID-19 from February 2020 to April 2020 were included in the study sample. We excluded patients who had negative RT-PCR for COVID-19 and those who were shifted to the intensive care unit (ICU) for mechanical ventilation.

Through our questionnaire, we collected demographics, performance status, comorbidities (including cardiovascular diseases, liver diseases, diabetes, and respiratory diseases), radiological features on presentations, escalation status, and the outcomes of the COVID-19. In-hospital mortality was calculated, and the patients were not followed up after their discharge from the hospital. The primary objective of this study was to identify the predictors of mortality in COVID-19 patients on CPAP during their hospital stay and compare it with patients who did not require CPAP during their disease course.

Microsoft Excel 2019®️ and IBM®️ SPSS®️ Statistics version 23.0 were utilized for data analysis. Data were manually entered into an Excel sheet and then transferred to SPSS through a comma-separated value (CSV) file, and on the basis of string and numerical values, variables were adjusted for further analysis. Central tendency and dispersion measurements were reported for continuous variables, while frequencies and percentages were calculated for descriptive variables.

The patients, who were grouped as those who were on CPAP and those who did not require CPAP (NCPAP), were cross-tabulated with various variables, including mortality/survival using the Chi-square test. To find out any potential relation between variables and mortality, a Pearson correlation test was also used. A p-value of ≤0.05 was considered significant.

## Results

A total of 56 patients diagnosed with COVID-19 (through RT-PCR) were included in the final analysis, among which 27 were on CPAP, while 29 did not require CPAP. The overall mean age of patients was 66 ± 14 (range: 26-94) years. The mean age of CPAP and NCPAP patients was 63 ± 15 (range: 26-85) years and 68 ± 13 (range: 40-94) years, respectively. The ethnicity of 54 (96.4%) patients was White-Caucasian, while 2 (3.6%) were British-Asian. In the study sample, 16 (28.6%) patients expired, of which 11 (40.7%) were on CPAP, while 5 (16.7%) did not require CPAP during the disease course; the ethnicity of all these patients was White-Caucasian. Table 1 shows important baseline characteristics of the participants.

**Table 1:** Baseline characteristics of the sample population ^1^Upper respiratory tract ^2^Lower respiratory tract ^3^Includes corticosteroids, biological agents, calcineurin inhibitors, inosine monophosphate dehydrogenase inhibitors, Janus kinase inhibitors, and monoclonal antibodies ^4^Angiotensin-converting enzyme inhibitors

Characteristic	Overall (N = 56)	NCPAP group (N = 29)	CPAP group (N = 27)
Age	66 ± 14	68 ± 13	63 ± 15
Gender			
Male	28 (50%)	14 (46.7%)	14 (51.9%)
Female	28 (50%)	15 (50%)	13 (48.1%)
Comorbidities			
Respiratory	27 (48.2%)	10 (33.3%)	17 (63.0%)
Cardiac	15 (26.8%)	7 (23.3%)	8 (29.6%)
Diabetes	15 (26.8%)	6 (20.0%)	9 (33.3%)
Hypertension	27 (48.2%)	15 (50.0%)	12 (44.4%)
Liver disease	6 (10.7%)	6 (20.0%)	-
Smokers	11 (19.6%)	1 (3.3%)	11 (40.7%)
Symptoms			
Fever	48 (85.7%)	23 (76.7%)	25 (92.6%)
Myalgia	22 (39.2%)	14 (46.7%)	8 (29.6%)
URT^1^	16 (28.5%)	10 (33.3%)	6 (22.2%)
LRT^2^	46 (82.1%)	23 (76.7%)	23 (85.2%)
Medications			
Antibiotics	26 (46.4%)	2 (6.7%)	24 (88.9%)
Immunosuppressants^3^	18 (32.1%)	15 (50.0%)	3 (11.1%)
Steroids	7 (12.5%)	1 (3.3%)	6 (22.2%)
ACE^4^ inhibitors	7 (12.5%)	1 (3.4%)	6 (22.2%)
Mortality	16 (28.6%)	5 (16.7%)	11 (40.7%)

The overall mean Medical Research Council Dyspnoea (MRCD) score was 2.54 ± 1.54, while it was 2.67 ± 1.56 and 2.41 ± 1.54 in the CPAP and NCPAP groups, respectively. The overall mean performance status (PS) for all patients was 1.57 ± 1.26, while it was 1.56 ± 1.18 and 1.59 ± 1.35 in the CPAP and NCPAP groups, respectively. On average, patients were unwell for 7.09 ± 4.68 days before the confirmed diagnosis of COVID-19, while patients stayed at home after the appearance of symptoms for 5.02 ± 5.6 days. In the CPAP group, this duration was 7.63 ± 4.1 and 8.63 ± 5.06 days, respectively. However, the mean average of unwell days before the COVID-19 diagnosis was 6.59 ± 5.18. Further statistical analysis failed to find any significant relationship between disease progression or survival and days of symptoms before the COVID-19 PCR test.

Correlation analysis showed that overall higher age, MRCD score, PS, previous liver disease, chronic use of angiotensin-converting enzyme (ACE) inhibitors, an increased respiratory rate on admission, and consolidation affecting more than one quadrant of the lungs were significantly correlated with increased mortality. Among patients receiving CPAP, higher age, MRCD score, and PS were significant predictors of mortality. Among the NCPAP group, advancing age, respiratory rate, MRCD score, PS, hypertension, liver disease, increased creatinine levels, and consolidation affecting more than one quadrant of the lungs were the predictors of mortality. Among the laboratory investigations, the increase in alkaline phosphatase (ALP) showed a positive relationship with mortality (r = 0.301, p-value = 0.024), whereas increased number of eosinophils showed a protective trend against fatality (r = 0.265, p-value = 0.49). Other variables, including neutrophils, lymphocytes, hemoglobin levels, CRP, platelets, and urea levels, did not yield any significant relationship. Table 2 shows a detailed correlation analysis of various mortality markers.

Chi-square analysis of predictive factors in relation to mortality depicted that, in our sample, survival in females was better than their counterpart gender. As shown in Table 3, a higher MRCD score, PS, and involvement of lung quadrants with consolidation increased the frequency of mortality. Any previous liver disease, chronic use of ACE inhibitors, or receiving palliative care also showed a trend of inclined mortality. However, patients who were placed under the “for full escalation” category of treatment escalation plan showed better survival than those who were receiving ward-based care.

**Table 2:** Correlation analysis of various variables with mortality outcome *Correlation is significant at ≤0.05. **Correlation is significant at ≤0.01.

Factors	Overall mortality in both groups (Pearson correlation)	Mortality in the NCPAP group (Pearson correlation)	Mortality in the CPAP group (Pearson correlation)
Age	0.376**	0.399*	0.486*
MRCD score	0.534**	0.596**	0.489*
Performance status	0.469**	0.486**	0.510*
Hypertension	0.181	0.441*	-0.017
Liver disease	0.292*	0.668*	-
Tachypnea	0.362*	0.443*	0.143
ACE inhibitors	0.359*	0.414*	0.282
High creatinine level	0.231*	0.714*	0.067
Consolidation of lung quadrants on CXR	0.292*	0.520**	0.016

**Table 3:** Distribution of participants based on CPAP and NCPAP grouping and survival

Characteristic	NCPAP patients (N = 29)^a^	CPAP patients (N = 27)^a^	Survived (N = 40)^b^	χ*, p-value	
Gender					
Male	14	14	18	0.72^ a^, 0.50	1.40^ b^, 0.188
Female	15	13	22		
MRCD score					
MRCD 1	14	8	22	7.18^a^, 0.126	20.9^b^, 0.001
MRCD 2	1	7	5		
MRCD 3	6	3	5		
MRCD 4	4	4	6		
MRCD 5	4	5	2		
Performance status					
PS 0	9	6	15	2.81^a^, 0.589	12.9^b^, 0.012
PS 1	6	8	11		
PS 2	3	6	6		
PS 3	10	6	7		
PS 4	1	1	1		
Consolidation in the number of quadrants					
Zero quadrant	12	4	14	6.79^a^, 0.149	4.88^b^, 0.299
One quadrant	2	3	4		
Two quadrants	7	5	9		
Three quadrants	4	6	6		
Four quadrants	4	9	7		
ICU step					
Full escalation	14	15	25	2.05^a^, 0.358	7.30^b^, 0.026
Step 2 RS	1	3	3		
Ward Care	14	9	12		

## Discussion

Our study aimed to identify risk factors in patients who were diagnosed with having COVID-19 disease requiring continuous positive airway pressure ventilation and factors leading to death while being on maximal possible treatment during those times.

Several studies have shown that, on admission, increased D-dimer concentration [7] and creatinine and cardiac troponin I [10] are all associated with a higher risk of failure of CPAP and mortality. In addition, a body mass index (BMI) of ≥35 kg/m^2^, increasing age [11], male sex, and comorbid status have been shown to be independently associated with worse in-hospital outcomes. Our study has shown some factors being contributive as above; increased MRCD score and involvement of more than one quadrant of the lungs are also being proportional to increased mortality.

One of the most feared complications of COVID pneumonia is progressive hypoxic respiratory failure, associated with dyspnea, tachypnea, and sometimes respiratory alkalosis [12].

This study has shown that the success of CPAP in COVID-19 patients was 59%, which was 26% higher as compared with the similar study done in Southend University Hospital, UK [13]. This could be attributed to the patient selection for management with CPAP. In addition, our study revealed that the mortality was slightly higher in males in contrast to the study by Noeman-Ahmed et al. [13], which did not show any gender difference.

Our study has shown that CPAP improves mortality in COVID pneumonitis, which is comparable to a previous study done by Walker et al. [14] in a similar setting like ours. They concluded that CPAP was beneficial in significantly reducing mortality. In addition, our study also highlighted the effect of pretreatment functional status, classified as per the WHO performance status, on the success of CPAP, an effect that has not yet been published to the best of our knowledge. However, the study done by Ashish et al. [15] has shown that care home residents with COVID pneumonitis had an increased risk of death. This presumably meant that poor performance status has an effect, but there was no clear documentation regarding the classification as per the WHO performance status scale.

While CPAP can be delivered outside the critical care setting, its effectiveness among patients with respiratory failure due to COVID-19 and ineligible for invasive mechanical ventilation is called into question by a few studies [16]. Our study provides further insight on to which cohort of patients with COVID pneumonitis is likely to benefit from CPAP. Hence, more multicenter studies involving a larger sample size are required to establish it.

The study of Thompson et al. [17] also shows comparable results to ours but with larger sample size. They confirm that death is more frequent with increasing age and in the White-British population. They also concluded that the most common comorbidities in their sample were hypertension at 46.4% and diabetes at 30.4%, compared with ours of 48.2% and 26.8%, respectively. The mortality rate in their study was 36%, compared with ours of 28%. Another interesting comparison was the presence of infiltrates involving more than 50% of the lung fields on admission chest radiograph was an independent predictor of death, which is also significant in our sample.

In another study by Chinnadurai et al. [18], the mortality rate was 40% with White Caucasian predominance of 87.4%, compared with ours of 100%. Moledina et al. [19] concluded that the most common symptom patients presented with was fever at 74.2%, compared with ours of 85.7%. Their analysis also showed increased creatinine as a predictor of mortality, which was confirmed by our correlation analysis.

Atkins et al. [20] had a mortality rate of 27.8%. The mean age of patients was 74 years, compared with ours of 66 ± 14 years. Again, 59.6% reportedly had hypertension as common comorbidity, although they do mention no positive correlation with death, which is contrary to our results. Elliot et al. [21] mentioned that the risk of death is higher in the older population, men, patients with comorbidities, and those taking ACE inhibitors, which is in keeping with our results.

The study of Knights et al. [22] was done at approximately the same time as ours, and their results showed a mortality rate of 31%. Of their patients, 24% required CPAP, and 21% did not survive, compared with ours of 48% and 40%, respectively. Again, they reiterate that age was a significant predictor of mortality. They also show data that confirmed promising results with CPAP, with 70% survival, compared with 60% in our study.

There were certain limitations of our study that can possibly have affected the results. Firstly, the sample size is small, and therefore, the findings cannot be generalized. Secondly, our data lacks a few vital variables that can affect mortality outcomes in COVID-19 patients, such as D-dimer levels and the weight of the patients. Measuring the clinical frailty score of elderly patients would have also helped us with better outcomes, and this might have confounded our results.

## Conclusions

During the initial phase of the COVID-19 pandemic (when we collected this data), the above findings from our study were novel. The results provided us markers that indicated the likelihood of success or failure of treatment, which help in the decision-making and rationalizing treatment during pressure period due to increased patient burden and limited availability of CPAPs. We understand that further studies are required to validate these markers as our sample size was not big enough compared with the prevalence of COVID-19.
